# Students’ feedback experiences and expectations pre- and post-university entry

**DOI:** 10.1007/s43545-022-00313-y

**Published:** 2022-01-29

**Authors:** Keith Gray, Robert Riegler, Michael Walsh

**Affiliations:** 1 Department of Economics, Finance and Accounting, Coventry University, Coventry, UK; 2 Department of Economics, Finance and Entrepreneurship, Aston Business School, Aston University, Birmingham, B4 7ET UK

**Keywords:** Higher education, Feedback expectation, Feedback experience, Feedback design

## Abstract

Feedback on submitted work is a crucial element of students’ learning process, but UK undergraduate students are often dissatisfied with the nature and process of university feedback. This article explores the causes of this dissatisfaction by analysing students’ past feedback experiences pre-university and how these relate to their expectations regarding feedback at the Higher Education level. This study is based on a large survey undertaken at a UK Business School at the beginning of course induction meetings for 1st year undergraduate students. The results reveal that there is a significant misalignment between students’ university feedback expectations and actual university feedback. These findings highlight the importance of managing student expectations regarding the nature and timeliness of feedback. This paper offers suggestions for feedback design and managing students' feedback expectations for course leaders to facilitate students’ transition from schools and colleges to UK universities.

**Supplementary Information:**

The online version contains supplementary material available at 10.1007/s43545-022-00313-y.

## Introduction

Transition from school to university can be a very challenging experience including many students leaving home for the first time, having to manage their personal finances, adapting to learn independently, but also reflecting on generic feedback on summative assessments that are returned weeks after submission deadlines. The outcomes from the annual UK National Student Survey (NSS) consistently demonstrate that undergraduate students are dissatisfied with the nature and process of university feedback (Williams et al. 2008)*.* This problem appears to be institution (Economics Network 2019) and subject discipline (Latreille 2010) wide. Thus, in order to improve students’ university experiences, it is important to understand students’ past feedback experiences and how these relate to their expectations regarding feedback at the Higher Education (HE) level.

This research explores the differences between pre-university feedback experiences and post-entry feedback expectations of undergraduates based in a large UK University Business School. Furthermore, this research discusses how the transition from schools and colleges to UK HE can be better facilitated through managing student expectations regarding the nature and timeliness of feedback. It is possible that expectations might affect student progression, development of transferable skills and academic outcomes, and employability.

Subsequently, the aims of the current research are as follows.

1. To explore the nature of student experiences prior to university entry in respect of feedback
2. To investigate university entrants’ expectations of feedback
3. To make a comparative examination of the data relating to feedback in respect of (1) and (2) above
4. To examine the implications of these prior aims for course leaders.

The sample covers three different programmes (Economics, Finance and Accounting), and consists of a large heterogeneous student body. The large sample and its variation allow us to generate a representative analysis for a UK Business School to meet the aims of this research project.

## Literature review

The literature on HE recognises that the transition from school to university can be difficult for students. There are a range of issues that make this transition difficult, including rising class sizes, a more heterogeneous student body (Hussey and Smith 2010; Adcroft 2011; Robinson et al. 2013; Jones et al. 2017; Money et al. 2020), universities’ commercialisation (Jones et al. 2017) and also limited contact time perceived as a lack of support (Beaumont et al. 2011).

The UK’s annual National Student Survey (NSS) typically indicates final year students are dissatisfied with assessment feedback, relative to other indicators therein (Jones et al. 2017; Nash and Winstone 2017; Carless and Boud 2018; Deeley et al. 2019; Lowe and Shaw 2019). In 2020, the outcome for assessment and feedback was 73%, contrasting with an overall satisfaction figure of 82% (NSS 2020). This relative discontent with assessment and feedback impacts negatively on retention rates, which is a particular concern for first year students during their transition to HE (Torenbeek et al. 2010). Low NSS assessment and feedback scores encourage HE institutions to focus on this area to subsequently improve NSS scores. Meanwhile, perceptions of feedback quality impact beyond the NSS. For instance, evidence suggests effective feedback can support independent learning (Winstone et al. 2017, Carless and Boud 2018) and develop transferable skills which enhance employability (Deeley et al. 2019). The literature suggests a mismatch between staff and students’ views of feedback (Muller and Tucker 2015, Winstone et al. 2017, 2021; Deeley et al. 2019) and implies the expansion of HE has widened the expectations gap between academics and students (Yorke and Longdon 2008; Adcroft 2011; Mulliner and Tucker 2017).

What is the role of students in the feedback process? Feedback was regarded as a teacher-centred information-transmission method where educators advise students. In this cognitivist model, the responsibility for feedback falls on academic staff transmitting information to students (Winstone et al. 2021, p. 119). Thus, students’ role in the feedback process is predominantly passive.

It is argued there is a greater need for ‘proactive recipience’ (Winstone et al. 2017, p. 18), where students engage in and take some responsibility for feedback to increase its effectiveness. Over the last decade, the literature has switched focus from the cognitivist model to a socio-constructive model (Winstone et al. 2021) where students take greater responsibility and where feedback may lead to further learning (Dawson et al. 2019). However, responsibility sharing needs further investigation (Nash and Winstone 2017), and it is not just engagement by staff or students, but both in partnership (Winstone et al. 2021). It is argued that in order to overcome barriers to student engagement with feedback greater staff responsibility is required for some of these barriers, whilst others require greater student responsibility (Nash and Winstone 2017).

Different feedback approaches in schools and HE partly explain the mismatch between school feedback experiences and university expectations. Feedback in schools is often teacher-centred whereby teachers offer students a structured and supportive environment (Money et al. 2020) and tend to give extensive feedback and guidance before work is submitted. Further, there is a focus on face-to-face feedback (pre-Covid-19 pandemic) and typically an emphasis on formative assessments (Jones et al. 2017). Contrastingly, the focus in HE is on independent learning and on summative assessments (Beaumont et al. 2011), due to greater student numbers and reduced contact time. Students lack preparation for independent learning (Jones et al. 2017) and thus feedback literacy needs to be developed (Carless and Boud 2018). Recent literature encourages a movement towards a more shared responsibility approach in universities (Winstone et al. 2021).

Universities have offered various solutions to reduce this expectation gap and ease the transition from school to HE. These range from measures to adjust student expectations, programmes to ease students’ transition (Smith and Hopkins 2005) and changing approaches to assessment/feedback. In terms of the latter, universities have adopted assessment practices to account for students’ prior experiences, such as enhancing pre-submission guidance (Beaumont et al. 2011) or making students more aware of what feedback is (Adcroft 2011).

There are several pertinent gaps in the feedback literature. Firstly, prior research focuses on HE and not pre-HE institutions by a ratio of 10:1 (Winstone et al. 2017). Secondly, there is a paucity of literature on preparing students for the transition to HE (Torenbeek et al. 2010). Meanwhile, authors emphasise the need for students entering HE to adapt from the structured learner approach at school to the more independent-learner approach (Money et al. 2020). Further research regarding the transition from school to HE could assist universities in helping students adjust, for instance through enhanced pre-induction activities focussing on independent learning and assessment (Murtagh 2012), and through re-designing the first year curriculum to embed such activities (Money et al. 2020). Thirdly, there is a specific paucity of studies which evaluate the link between students’ feedback experiences and expectations pre- and post-university entry. This study attempts to address this problem. In contrast to previous studies which investigated sixth formers’ expectations of university feedback (Smith and Hopkins 2005; Jones et al. 2017), or teachers’ perceptions of transition (Money et al. 2020), the current research focuses on students who progressed to university and were surveyed at the point they arrived, before they received any induction information to minimise the respondents’ bias regarding feedback expectations.

## Methods and sample description

This study focuses on feedback expectations of students at the beginning of their university journey. Feedback can take many different shapes, e.g., verbal feedback to the whole class or to individuals, written generic or individual comments, videos, etc. For this research, we were especially interested in students’ expectations of feedback on a submitted written assessment, e.g., an in-class test, coursework essay or exam.

The research was conducted at a large Business School in a UK university. The research evidence was gathered by developing and distributing a paper-based survey questionnaire to first year undergraduate students attending their subject specific Course Induction Meetings (CIM) in the autumn of the academic year 2016/17. The surveys were completed at the CIM as the students’ first formal activity and before course information was provided by course teams, in order to limit the impact of bias in their expectations related responses.

The survey questionnaire was adapted from that of Yorke and Longdon (2008) to meet the aims of the current research. It focused upon background data (including gender, ethnicity and first in family to university), issues regarding time spent in class, with feedback being of primacy. The survey questions comprised a mix of closed, dichotomous and Likert-style questions.

Whilst the survey was comprehensive, it was designed to be completed in fifteen minutes to minimise the risk of questionnaire fatigue and thus maximise the response rate. Written student agreement to participate was sought and students received a summary of the aims of the research.^1^ The questionnaire was completed anonymously and all data stored securely in line with the University and UK’s data protection protocols.

In total, the distribution of students enrolled on three first year undergraduate courses for 2016/17 was as follows (Table 1):

With 62–63%, the response rate was similar over all courses. All students that attended the CIM completed the survey. Students enrolling late or not attending their CIM were excluded. There was a potential for sample-selection bias if non-attendance was a non-random event, e.g., if overseas students and late enrollers had different feedback experiences. Despite this, excluding such students ensured that student responses were unaffected by information about feedback provided post CIM.

**Table 1:** Number of students enrolled on a programme at a large UK Business School, the sample size of students participating in this study and the relative response rate (in %)

Undergraduate course	Numbers of students enrolled	Number of participants	Response rate (%)
Economics	272	169	62.1
Finance	130	82	63.1
Accounting	278	174	62.6
Total	680	425	62.5

The data of the three different courses were aggregated and analysed. This aggregation produced more variation in the sample and, as a result, led to a more representative study for the whole Business School at this case study university.

Table 2 shows that approximately 37% of the sample identified themselves as female students. The student body was very diverse with respect to ethnicity, i.e., around 31% of students were Asian (excl. Chinese), 31% of white and 22% of black ethnicity. The majority of students (68%) entered university via the A-levels route, and around 35% of students are the first within their families to go to a university.

**Table 2:** Number and share of students with respect to selected students’ characteristics

Responses	Number	Share (%)
	425	100
**Gender**		
Identified as male	266	62.6
Identified as female	155	36.5
No response	4	0.9
**Ethnicity**		
White	132	31.1
Black or Black British	93	21.9
Asian or Asian British	133	31.3
Chinese	42	9.9
Other	20	4.7
No response	5	1.2
**A-levels**		
Yes	289	68.0
No	122	28.7
No response	14	3.3
**First to go to University**		
Yes	149	35.1
No	270	63.5
No response	6	1.4

**Fig. 1:**
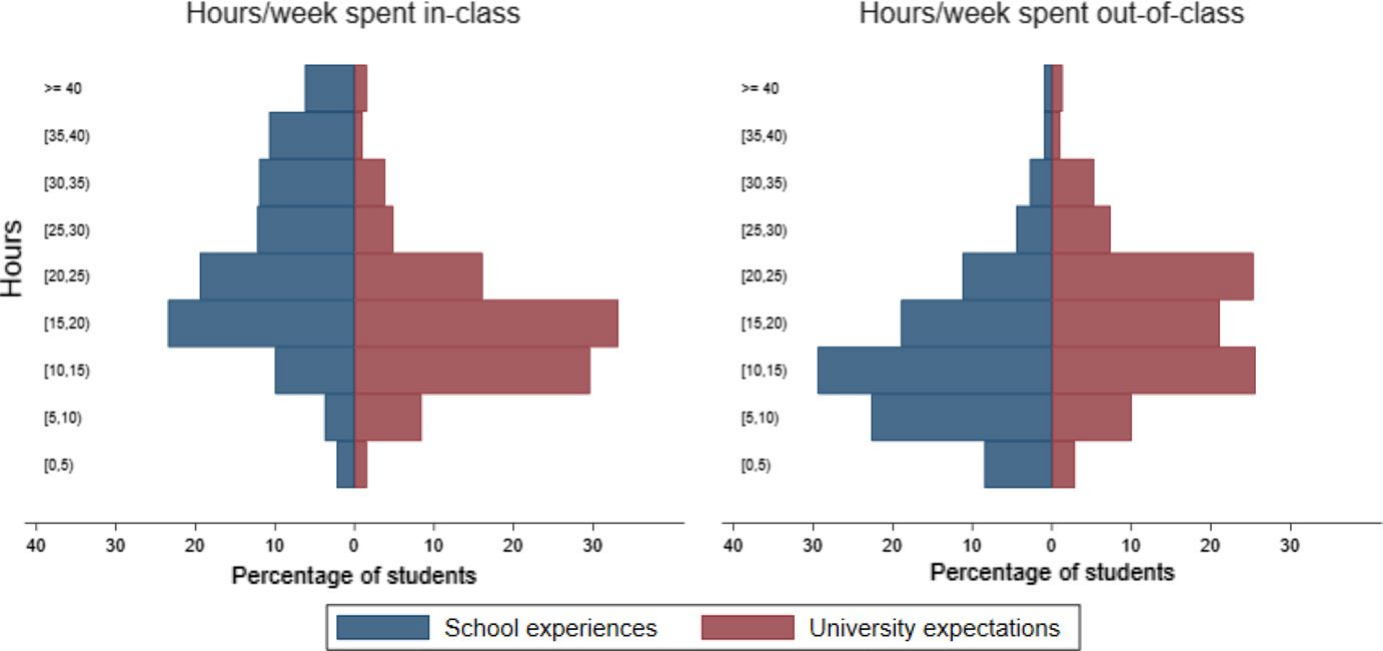
School experiences and university expectations about the average hours per week spent studying in-class (left panel) and out-of-class (right panel)

**Fig. 2:**
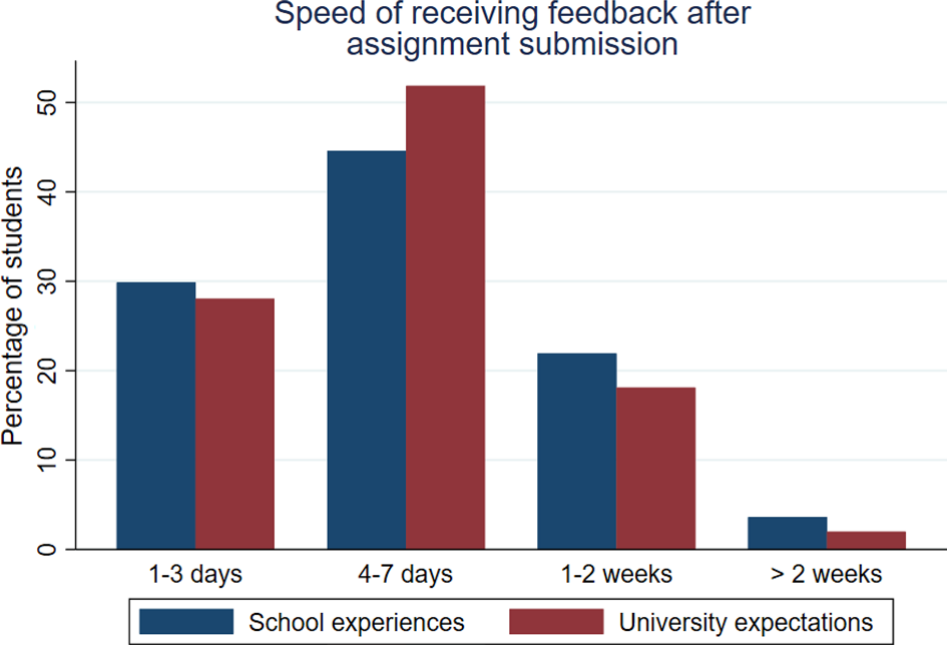
School experiences and university expectations about average feedback turn-around after assignment submission

**Fig. 3:**
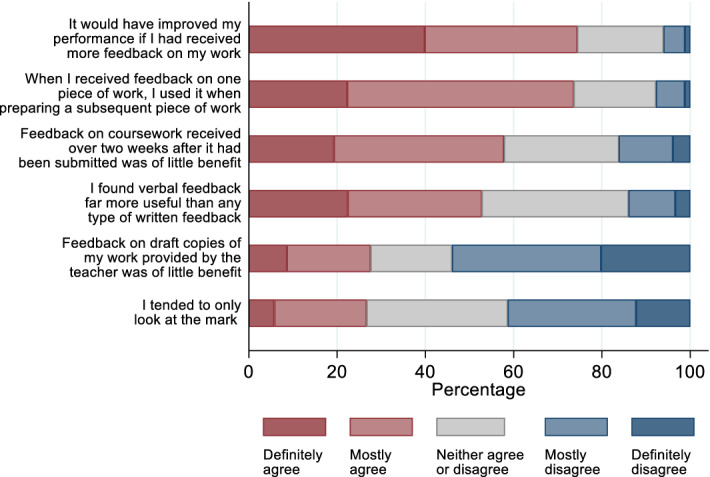
Students’ feedback experiences in school

**Fig. 4:**
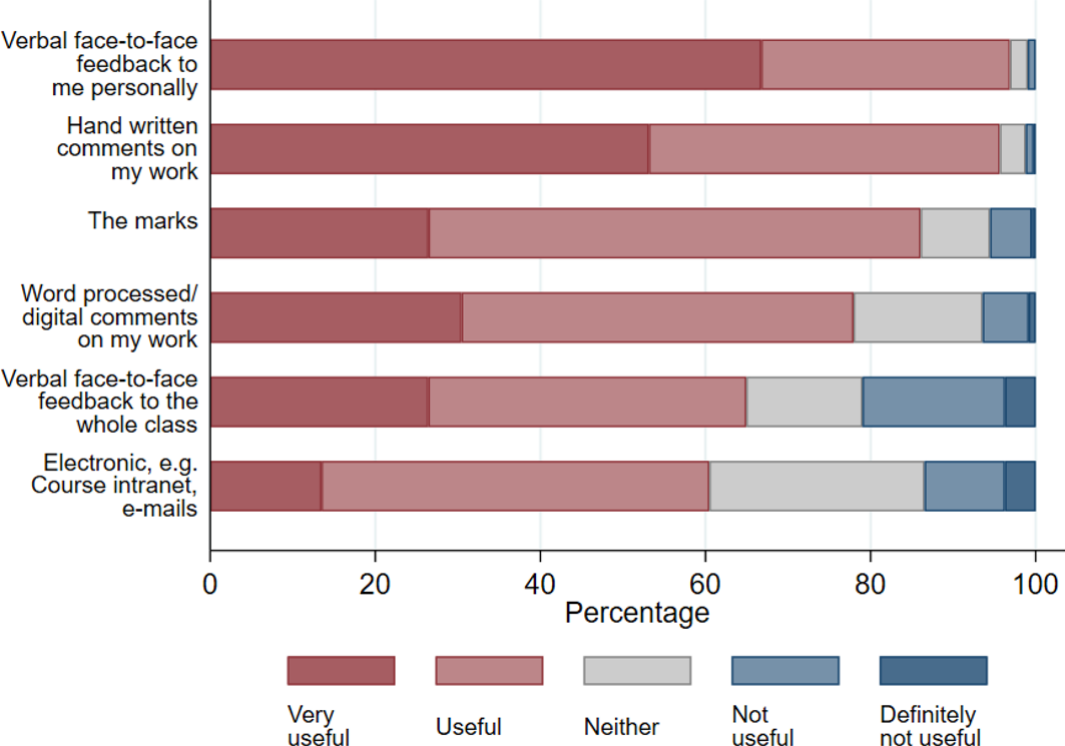
Perception of usefulness of different feedback types received at school

**Fig. 5:**
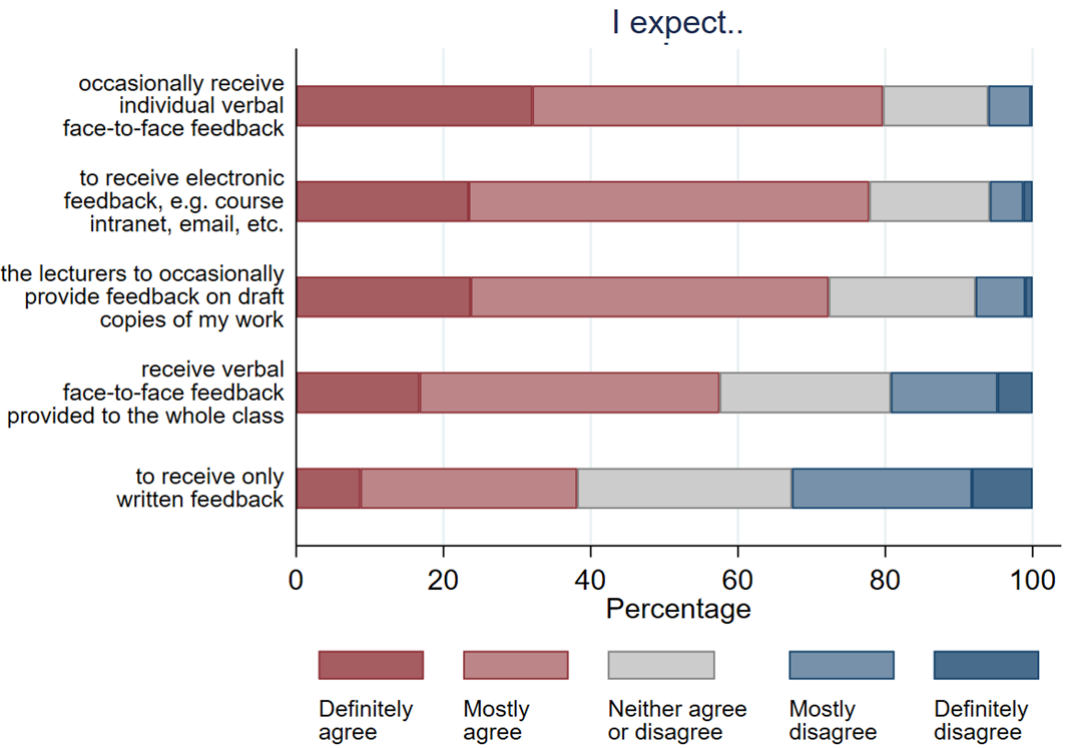
University expectations about types of feedback to be received at university

The results of the survey were summarised using descriptive statistical techniques. For numerical variables, paired sample *t* tests were undertaken to test if the differences between school experiences and university expectations were statistically significant. For ordinal variables, a Wilcoxon signed-rank tests was employed instead. All survey questions that were used in this study and shown in Figs. 1, 2, 3, 4, 5 are presented in Table A1 in the Online Appendix.

## Results and analysis

### Learning environment

The literature review offered evidence that the transition from school to university might be difficult for students. One factor that exacerbates transition are differences in the learning environments at school and at university, e.g., average class sizes in HE Business Schools may be larger than what students experienced pre-university entry. Thus, we initially explored comparative differences between the learning environment prior to and students’ expectations on arrival at university.

The blue bars in Fig. 1 (left panel) above show the distribution of average hours per week students spent in-class at school. Even though a peak can be observed in the range of 15–20 h (23%), the majority of students (60.1%) spent more than 20 h in-class. The change in the shape of the distribution of university expectations (red bars) evidenced that many students adopted their expectations on arrival at the University. Only a few students expected more than 25 h in-class, and 73% of students expected less than 20 h. On average, students expected to spend 7 h per week less in-class at university.^2^ A two-tailed paired sample t-test showed that the difference between students’ expectations about hours spent in-class at university and their experiences of hours spent in-class at school was statistically significant [*t*(379) = 14.2, *p* < 0.001].

To become independent learners, it is important for students to study in their own time out-of-class. Figure 1 (right panel) above compares students’ prior experiences and their expectations about how many hours they expected to learn independently. There are significant differences in the distributions, showing that students had an understanding of the importance of learning out-of-class. A small group of students expected to work less than 10 h on their own at university (13%), a percentage that is lower than at school (31%). On average, students expected to spend 4 h per week more studying out-of-class. Using a two-tailed paired sample t-test, the difference between students’ expectations about time spent on studying outside the classroom at university and their experiences of time spent on studying outside the classroom at school was statistically significant [*t*(371) = − 9.89, *p* < 0.001]. Thus, students’ expectations about out-of-class learning reflected the reality.

It appeared that students had generally a good idea about the learning environment at university. Statistical results showed that students’ experiences and expectations were significantly positively correlated for in-class studying (*r* = 0.45, *p* < 0.001) and out-of-class studying (*r* = 0.58, *p* < 0.001) indicating that experiences were related to expectations. However, when asked about expectations related to more module specific issues, a more significant misalignment of expectations was observed. For example, when asked how often work has to be submitted in a core module, the majority of students (51%) expected to submit work at least once a week and only 2% expected to submit work only once a semester.

### Feedback

This section presents the findings related to feedback and explores to what extent students have realistic expectations about university feedback.

#### General feedback experiences and expectations

Figure 2 above compares how quickly students received feedback on an assignment after they had handed it in at school (blue bars) with their expectation on how quickly they will receive feedback at university (red bars). There was little difference, which is in accordance with findings mentioned in Jones et al. (2017). The majority of students were used to receiving feedback within 7 days (74%) and the majority still expected the same turn-around at university (80%).^3^ A Wilcoxon matched-pairs signed-rank test did not find any statistically significant median pair-wise differences between students’ experiences and expectations (*z* = 0.731, *p* = 0.47). This result implies that, in contrast to before, students did not adjust their university feedback expectations correctly. Only 2% expected to wait more than 2 weeks for their feedback at university. This result could explain the relatively disappointing NSS results at the case study university and the wider HE sector with respect to feedback. Students’ expectations were not in-line with the protocols on feedback turn-around times, especially for large modules at the case study university, leading potentially to negative perceptions of the learning experience.

The majority of respondents (58%) regarded feedback given 2 weeks after submission as of little benefit (Fig. 3). This reinforces why actual experience regarding feedback time post-entry to university will potentially negatively affect the quality of the learning experience.

Furthermore, the majority of respondents, some 74% placed importance on the ‘volume’ of feedback—this raises a wider problem for course leader and is considered in the section on implications for course/assessment design provided later in this paper. Also of note, 74% of respondents claimed they used prior feedback to enhance future assessment performance. However, understanding and acting on feedback is not an easy task and will require support to develop their feedback literacy (Carless and Boud 2018).

#### Experiences and expectations about different feedback types

This section presents the results of students’ perception on the usefulness of different feedback methods they have experienced at school and is followed by a discussion about university feedback expectations.

The data presented in Fig. 4 above indicate that individuality of feedback appeared to be an important determinant for students’ perception of the usefulness of feedback in school. Some 97% of respondents found verbal face-to-face feedback to be most useful, followed by hand-written comments with 96%. Around half of the students (53%, Fig. 3), highlighted that they thought that verbal feedback is far more useful than written feedback. Even though verbal feedback was perceived as most useful, it also was one of the most resource intensive methods of feedback. For modules with several hundreds of students, it may not be feasible. General verbal feedback to the whole class could be seen as an alternative; however, the questionnaire results also revealed that students experienced it as far less useful. Technology can provide an opportunity to provide voice recorded feedback on online exams and coursework to generate a more personal feedback experience.

Notably, whilst 96% of respondents found handwritten comments useful/very useful, only 78% felt as strongly regarding digital comments. There are several explanations for latter finding: e.g., respondents may prefer ‘humanised’ or personal input (i.e., handwritten comments) because they perceive the tutors’ efforts in providing the feedback have been more time consuming and therefore are more important. However, there may be a plethora of other explanations. These include that respondents may have received more written feedback than digital comments at school and thus prefer written feedback that resembles their typical learning experience at schools.

Meanwhile, 86% thought that the marks were a good method of feedback, but only around a quarter of students (27%) tended to look only at the mark rather than any other forms of feedback (see Fig. 3). This result is encouraging as a mark only has a limited potential in providing students with an indication on how to improve their work in upcoming assessments. However, 27% still ignored the array of alternative types of feedback indicating a potential lack of feedback literacy skills. Finally, feedback via emails and announcements on the module web were perceived as less useful.^4^

Although not possible in the context of the current research, it would be interesting to consider how student preferences for personalised or one-to-one feedback are affected by the greater use of remote learning as required during the Covid-19 pandemic. With the recent move to online teaching at HE institutions, it is likely that students are far more used to digital feedback than before the Covid-19 pandemic.

In Fig. 3, only 30% definitely or mostly agreed that feedback by tutors on drafts was of little benefit whilst by contrast over 50% mostly or definitely disagreed. This is notable because it implies a latent demand for draft feedback from tutors when crafting final assessment submissions. This highlighted an important mismatch between the expectations of student respondents and the reality of academic workloads within the case study university, which prevented regular provision of feedback on draft assessments. Furthermore, this implies that students may over-relied on tutor (‘guru’) input in preparing for final assessment submissions. Such reliance is at odds with the latest thinking on the importance of peer-group feedback and reflective/self-critical assessment (Nicol et al. 2014; McConlogue 2015; Guest and Riegler 2017, 2021; Carless and Boud 2018) both of which are key elements of employability as well as academic learning development. It is clear that both university academic and careers/employability staff will need to help students transform their expectations regarding access to individualised draft feedback. Of course, it is noted that the respondents’ expectations were pre-determined by their experience of and exposure to the imposed pedagogic practice at the secondary stage of their education.

The most interesting points raised related to feedback are that around 80% of students expected to receive verbal face-to-face feedback occasionally (see Fig. 5). However, this was traditionally very unlikely in the first year of students’ undergraduate studies. Verbal face-to-face feedback by the tutor was often only received in office hours for a small number of students given the resource constraint faced at the case study university. Recognising they have enrolled on a large course may explain why around 58% of respondents expected to receive verbal feedback provided to the whole class. However, peer feedback could be used to provide personalised verbal face-to-face feedback even on large courses (Wei et al. 2020).

Meanwhile, 40% definitely/mostly agreed they will only receive written feedback. Arguably, good practice in the design and delivery of feedback in HE requires a much broader/integrated approach to feedback on summative assessments—for instance, including group wide audio, video and written digital feedback via learning platforms such as Blackboard, Moodle and Aula. Also of note, almost three quarter of students expected feedback on draft copies of academic work—an unrealistic expectation on large courses.

## Implications for course leaders regarding feedback

Our results suggest that students’ project their school experiences about feedback onto their university expectations. The findings identified a number of areas of feedback provision and practice where such a misalignment will need to be tackled. Further, the literature identifies that such misalignment cannot be ignored because of its impact on students’ performance and overall student satisfaction (Winstone et al. 2017, 2021; Deeley et al. 2019). Additionally, the authors recognise that responsibility for realignment of expectations falls both upon students and university academics, i.e. a shared responsibility approach should be adopted (Nash and Winstone 2017; Winstone et al. 2021). In what follows, the authors reflect upon this paper’s key findings and the relevant academic literature on feedback to identify how course leaders can assist students in closing the misalignment between students’ expectations of the university’s feedback model and the reality.

With 80% of respondents expecting feedback with a seven-day period and just 2% expecting to wait more than two weeks (Fig. 2), this has important implications for managing student expectations. Firstly, course leaders need to identify and communicate from day one what the specific university practices are on turnaround times for summative assessments. Indeed, given the growing importance of bridging type exercises^5^ provided by universities pre-entry, efforts could be made to manage student expectations regarding such turnaround times pre-course starts. Secondly, the evidence implies the opportunity at course entry to indicate to students with greater emphasis the differences between summative and formative assessment and underline the protocols surrounding feedback times on each of these. Formative feedback occurs at multiple points and is often speedier: examples include in-class solutions, personal tutorials, casual conversations walking across campus, and even email responses (Lowe and Shaw 2019). Thus, course leaders and fellow tutors can help students recognise the broad spectrum of continuous feedback opportunities arising during their learning journey and help re-align students’ expectations regarding feedback timelines.

A further challenge faces course leaders: evidence shows that 58% of respondents stated that feedback on assessments received over two weeks after submission was of little benefit (see Fig. 3). This result is in-line with findings by Deeley et al. (2019) that feedback in assessments becomes irrelevant from a student’s perspective if not received in a timely manner. This is concerning and represents a paradox vis-à-vis the respondents’ time preference and quality trade off regarding feedback. In terms of the former, and on large courses like those provided in the case university, a key pressure point is staff meeting protocols for the return of marked assessments. The case university operates a ten working day turnaround protocol for undergraduate Level 6 (finalists), and of more concern given the evidence, fifteen working days for Levels 4 and 5. Other UK institutions will have similar marking turnaround times. This implies that during the first year of transition into university life, a large number of students will have negative impressions of the usefulness of their feedback. Meanwhile, paradoxically, students will trade-off a delay in the speed with which feedback is returned in exchange for higher quality, in-depth feedback (Ferguson 2011). This paradox is observed within the case university’s NSS, and institutional course and module evaluation data.

Evidence from the current survey identified that 74% of students think that if they had received more feedback on assessments in school, they would have been able to perform better (see Fig. 3). This provides an opportunity in two ways. Firstly, course leaders and module leaders when designing feedback models can identify a best practice ‘minimum’ of feedback, taking account of the trade-off between feedback quantity and quality. Secondly, in large schools within universities (more than 100 members of academic staff), following Robinson et al. (2013) and Deeley et al. (2019), marking teams can develop feedback practices which enable standardisation and emotional reassurance (i.e., a balance of positive and negative feedback).

Only 30% of respondents definitely or mostly agreed that feedback on draft assessments was of limited benefit (see Fig. 3)—thus there is an implicit assumption at the point of entry to university that respondents value feedback on draft work. This evidence provides course leaders with an opportunity for staff development—tutors can be encouraged to develop exercises and activities designed around their assessments, which provide feedback to feed-forward prior to the submission of summative assessments. One great advantage of this is that any misunderstanding by students regarding the nature, purpose and evaluation of their summative submissions can be identified and rectified. This would beneficially enable students to perceive the process of academic support regarding their performance in assessments as a continuous one rather than an ex-post event—this is of merit, given the experience of the current authors that students often treat feedback on summative assessments as merely an act of ‘grade revelation’.

Arguably the current learning environment facing students and universities regarding the Covid-19 pandemic provides universities with an opportunity to pursue other avenues for feedback provision. Specifically, the widespread availability of digital media such as Microsoft Teams, Zoom and others, enables tutors to develop a revised mind-set regarding how, when and in what format feedback should occur. For instance, the flexibility of Microsoft Teams enables tutors to invite students or small groups of students to scheduled, brief or ad hoc meetings. This enhances the variety of ways students then receive feedback and guidance on formative assessments and provides additional input to the development of skills required for summative assessments.

## Further research and study limitations

This large-scale study collected and analysed information about students’ feedback experiences and university expectations at a large UK Business School. The results showed that students had quite accurate expectations about the university learning environment, but with respect to feedback and feedback turn-around, the expectations were rather inaccurate. Future research should undertake focus-group meetings to explain to what extent feedback expectations are linked to school experiences and what other factors are shaping them. Furthermore, the survey related to Fig. 3 could be considered as ‘leading’, therefore creating a bias in the results. Specifically, the share of participants who agree with an assertion can be different to the share of people who disagree with the opposite assertion (Krosnick and Presser 2010). Extensions to the current research will use more neutral wording or also include opposing questions. However, more questions may come at the cost of questionnaires becoming more extensive, therefore increasing the risk of questionnaire fatigue.

## Conclusions

Using a large-scale survey, this study (i) analysed the nature of student experiences prior to university entry in respect of feedback, (ii) investigated university entrants’ expectations of feedback, (iii) undertook a comparative examination of feedback experiences and expectations, and (iv) discussed the implications of the results for course leaders. Whilst the study found that students had accurate expectations of the general HE learning environment, we could not find similar accuracy regarding expectations about university feedback, e.g. only a small minority of students expected to wait for feedback on a submitted assignment for more than two weeks. The majority of students also indicated that feedback provided after 2 weeks from the submission is of little benefit, highlighting a potential reason why students do not rate university feedback highly. Efforts to manage student expectations regarding such turnaround times prior to the start of the course could mitigate students’ disappointment.

Verbal face-to-face feedback and written comments were both perceived as very useful. A majority of students expected to receive occasional verbal face-to-face feedback, which can be provided in personal meetings during office hours. However, this face-to-face feedback provision is not feasible for large modules due to limited supply of office hours slots (in-person or online). Similarly, the majority of students expected to receive written comments on draft copies of their work which is also not easy to resource on large modules. Shifting students’ expectations towards formative feedback during seminars, webinars, and other informal channels outside the classroom could be a panacea. Tutors should be encouraged to develop exercises and activities designed around their assessments, which provide feedback to feed-forward prior to the submission of summative assessments. This would enable students to perceive the process of academic support as a continuous rather than an ex-post event and will spread their workload more evenly across the academic year.

## Supplementary Information

Below is the link to the electronic supplementary material.

Supplementary file1 (DOCX 18 KB)

## Data Availability

The datasets generated during and/or analysed during the current study are available from the corresponding author on reasonable request.

## Footnotes

1 Ethical approval for this research was obtained from the University Ethics Committee. Before the survey was distributed, students were informed about the study and participation. Students had the opportunity to withdraw consent before and after the survey took place. All data collected were anonymous.

2 Detailed descriptive statistics can be found in Table A2 in the Online Appendix.

3 A *Χ*^*2*^-test showed that there is a highly statistically significant relationship between school experiences and university expectations [*Χ*^*2*^(4) = 48.1, *p* < 0.001]. To ensure a valid test result, the categories “1–2 weeks” and “more than 2 weeks” had to be merged.

4 Feedback does not only have to be provided by lecturers but can also involve peer-evaluations. Peer-evaluations reduces the problem of resource constraints and generates personalised feedback. However, with just 50%, we found that it was perceived as the least useful feedback method.

5 E.g., collecting economics artefacts that can explain real world phenomena.
