# Supplementary material for: Students’ feedback experiences and expectations pre- and post-university entry
Source: SN Soc Sci. 2022 Jan 29;2(2):16. doi: 10.1007/s43545-022-00313-y (PMC8800546; doi:10.1007/s43545-022-00313-y)
Supplement: Supplementary file 1 — Supplementary file1 (DOCX 18 KB) [file 43545_2022_313_MOESM1_ESM.docx]

| **Figure 1** | |
| --- | --- |
| Question | Please indicate the average number of hours you spent in the classroom at school/college each week. |
| Options | - |
| Type | Numeric |
| Question | Please indicate the average number of hours you would expect to spend in the classroom at university in a typical week. |
| Options | - |
| Type | Numeric |
| Question | Please indicate the average number of hours you spent studying outside of class/school each week. |
| Options | - |
| Type | Numeric |
| Question | Please indicate the average number of hours you would expect to study outside of class at university in a typical week. |
| Options | - |
| Type | Numeric |
| **Figure 2** | |
| Question | Typically, how quickly did you receive feedback on a piece of work after you had handed it in? |
| Options | 1-3 days; 4-7 days; 1-2 weeks; > 2 weeks |
| Type | Categorical |
| Question | How quickly would you expect to receive feedback on a piece of work after you have submitted it at university? |
| Options | 1-3 days; 4-7 days; 1-2 weeks; > 2 weeks |
| Type | Categorical |
| **Figure 3** | |
| Question | Please indicate the extent to which you agree with each statement below. |
| Options | - It would have improved my performance if I had received more feedback on my work - I tended to only look at the mark I tended to only look at the mark - When I received feedback on one piece of work, I used it when preparing a subsequent piece of work - I found verbal feedback far more useful than any type of written feedback - Feedback on draft copies of my work provided by the teacher was of little benefit |
| Type | Likert scale: Definitely agree; Mostly agree; Neither agree or disagree; Mostly disagree; Definitely disagree; N/A |
| **Figure 4** | |
| Question | Which of the following types of feedback did you find the most useful? |
| Options | - The marks - Handwritten comments on my work - Word processed/digital comments on my work - Verbal face-to-face feedback to the whole class - Verbal face-to-face feedback to me personally - Electronic, e.g. Course intranet, e-mails |
| Type | Likert Scale: Very useful; Useful; Neither; Not useful; Definitely not useful; I have not received feedback in this way |
| **Figure 5** | |
| Question | Please indicate the extent to which you agree with each statement below: |
| Options | - I expect to receive only written feedback - I expect to receive feedback via online (digital) sources e.g. Moodle, Blackboard, email, etc. - I expect to receive verbal face- to -face feedback provided to the whole class - I expect to occasionally receive verbal face- to- face feedback provided to me individually - I expect the lecturers to occasionally provide feedback on draft copies of my work |
| Type | Likert scale: Definitely agree; Mostly agree; Neither agree or disagree; Mostly disagree; Definitely disagree; N/A |

Table A1 – Survey questions used in this study and presented in Figures 1 to 5

| **Variable** | **Obs** | **Mean** | **Std. Dev.** | **Min** | **Max** |
| --- | --- | --- | --- | --- | --- |
| School experiences: in-class | 401 | 22.6 | 9.6 | 2 | 56 |
| Uni. expectations: in-class | 392 | 15.9 | 7.1 | 2 | 60 |
| School experiences: out-of-class | 401 | 12.9 | 7.5 | 0 | 40 |
| Uni. expectations: out-of-class | 379 | 16.5 | 7.9 | 2 | 60 |

Table A2 – Descriptive statistics for students’ school experiences and university expectations on hours per week spent in-class or out-of-class studying
